# Gain Control Network Conditions in Early Sensory Coding

**DOI:** 10.1371/journal.pcbi.1003133

**Published:** 2013-07-18

**Authors:** Eduardo Serrano, Thomas Nowotny, Rafael Levi, Brian H. Smith, Ramón Huerta

**Affiliations:** 1 GNB, Escuela Politécnica Superior, Universidad Autónoma de Madrid, Madrid, Spain; 2 CCNR, Informatics, University of Sussex, Brighton, United Kingdom; 3 Department of Neurobiology and Behavior, University of California, Irvine, California, United States of America; 4 School of Life Sciences, Arizona State University, Tempe, Arizona, United States of America; 5 BioCircuits Institute, University of California San Diego, La Jolla, California, United States of America; Indiana University, United States of America

## Abstract

Gain control is essential for the proper function of any sensory system. However, the precise mechanisms for achieving effective gain control in the brain are unknown. Based on our understanding of the existence and strength of connections in the insect olfactory system, we analyze the conditions that lead to controlled gain in a randomly connected network of excitatory and inhibitory neurons. We consider two scenarios for the variation of input into the system. In the first case, the intensity of the sensory input controls the input currents to a fixed proportion of neurons of the excitatory and inhibitory populations. In the second case, increasing intensity of the sensory stimulus will both, recruit an increasing number of neurons that receive input and change the input current that they receive. Using a mean field approximation for the network activity we derive relationships between the parameters of the network that ensure that the overall level of activity of the excitatory population remains unchanged for increasing intensity of the external stimulation. We find that, first, the main parameters that regulate network gain are the probabilities of connections from the inhibitory population to the excitatory population and of the connections within the inhibitory population. Second, we show that strict gain control is not achievable in a random network in the second case, when the input recruits an increasing number of neurons. Finally, we confirm that the gain control conditions derived from the mean field approximation are valid in simulations of firing rate models and Hodgkin-Huxley conductance based models.

## Author Summary

Neural networks in the brain can classify objects as being the same thing regardless of the stimulus intensity, which is referred to as gain control. This intensity invariance occurs during pattern recognition in any sensory modality. We evaluate whether it is possible to design stable neural circuits made of excitatory and inhibitory neurons that are capable of controlling the internal representation of a stimulus using network properties alone. Gain control is important because if the activity gets out of control neurons can die or be damaged by hyper-excitation. It is known that one can control the internal representation by the saturating responses of neurons. However, we show that there also is a precise relationship of network parameters that can account for gain control regardless of the external stimulus without such saturation. The most important network parameters are the connections from the inhibitory population to the rest of the network. This is consistent with experimental findings. We also show that the connections from the excitatory to the inhibitory population do not play an important role in gain control, suggesting that they can be freed for encoding purposes without leaving the operating range of the network when levels of stimulation increase.

## Introduction

In sensory perception the salient properties of signals need to be separated from their overall amplitude and, therefore, at some level in the neural processing cascade the response of neurons should become insensitive to the overall amplitude of stimulation. This is the role of gain control, which is ubiquitous for sensory processing in the brain [1]. It allows us to recognize a melody independent of how loud the music plays, identify objects in a wide range of light conditions or recognize an odorant irrespective of its concentration or our distance from the source [2].

In the olfactory system it is particularly important to distinguish odorant composition from intensity. A foraging moth or bee can visit many flowers in a day. During their foraging trips the intensity of stimulation fluctuates over a wide range of concentrations while they approach or leave their target flowers. Nevertheless, they are able to discern a preferred odor and reach their goal, consistent with perceiving the odor at different concentrations as a single perceptual object [3], [4]. This is a typical example where adjusting the organism's sensitivity by setting the appropriate sensory gain for environmental cues is critical for matching the animal's behavioral responses to its ecological needs.

Odor encoding is a spatially distributed process. Olfactory receptor neurons (ORNs) in the antennae detect odors and relay neural activity to the antennal lobe (AL). In insects, each ORN typically expresses two odorant receptor genes. One is ubiquitously expressed in all ORNs. The other is unique to subpopulations of ORNs [5], [6]. The unique receptor determines the range and intensities of odors that the ORN detects. ORNs expressing the same receptor protein send axons onto a single glomerulus in the AL [7], the first signal processing stage of the olfactory pathway. Thus, each odorant receptor gene defines a processing channel which carries information about some particular feature of the odorant stimulus. The stereotypic organization of the AL is relatively simple. Each glomerulus is innervated by about three to five uniglomerular projection neurons (PNs) which propagate the olfactory information downstream to higher brain centers. Glomeruli are anatomically connected by a network of local interneurons (LNs). The AL has both excitatory (eLN) and inhibitory (iLN) local neurons [8]–[10] and inhibitory local circuits play an important role in shaping the response of the output [11], [12]. LNs exclusively branch within the AL and therefore provide a substrate for interactions between olfactory channels.

At the first level of olfactory perception, insects are sensitive to concentration. The stronger the odor the stronger the excitation that the PNs receive and, in addition, the more glomeruli are recruited [13]–[16]. However, by recording the activity of more than 100 PNs it has been found in locusts [17] that the mean firing rate of the excitatory PN population in the AL remains nearly constant across a large range of odor concentrations. Subsequent intracellular studies of the excitatory neurons in *Drosophila* have confirmed these results [18] by identifying a nonlinear transformation that saturates the PN response to the ORN activation, effectively creating a situation where the level of activity of PNs is insensitive to changes in intensity. Independent evidence in bees indicates that the lateral antenno-cerebral tract (lACT), one of the olfactory pathways in bees, similarly shows very low sensitivity to concentration [19] and is therefore also likely to be subject to gain control. It has also been established using simultaneous optical and electrophysiological recordings in several glomeruli of the *Drosophila* antennal lobe that the PNs reach their maximum firing rate in response to various odorants at intermediate concentrations [20]. It is this regime prior to full saturation of neural responses at very high concentrations that we are addressing in this paper.

Theoretically one can explain the need for gain control in the AL [21], [22] because the next processing layer of the olfactory system, the mushroom bodies, display sparse activity [23], [24] which is a critical feature of models of associative memory [25]–[27]. But sparse coding is also very sensitive to fluctuations in input strength [21], [28]–[30] implying that the level of activity in the AL has to be carefully controlled.

A number of studies has illustrated the importance of lateral inhibitory networks for sharpening the tuning curves of PNs in response to odors [8], [11], [13], [31]–[33] and demonstrated their role in the formation of odor-specific spatio-temporal activity patterns in the AL [34]–[40]. In particular in [40] the authors analyze how lateral inhibition normalizes the response of a PN to its presynaptic ORNs and how this type of gain control affects PN population codes for odors in *Drosophila*. In addition, it has been shown in a model of the bee olfactory system [41] that explicitly added gain control allows improved coding of odors and odor mixtures. In this work we analyze the structural and functional network requirements that lead to gain control that keeps the excitatory neurons within a defined narrow range of activity regardless of the stimulus intensity.

**Figure 1:**
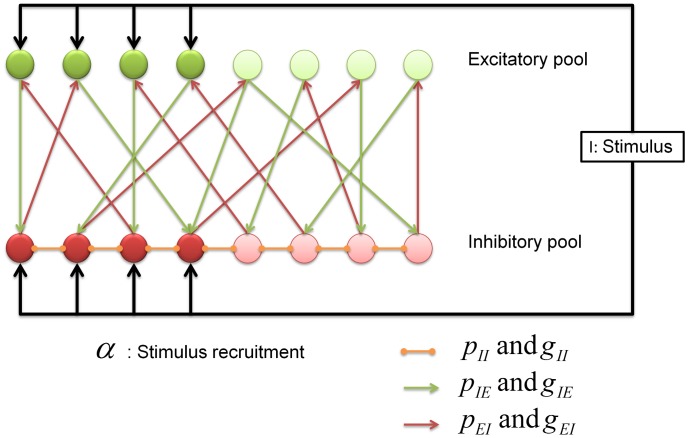
Schematic representation of the network architecture. There are two populations of neurons, excitatory (green) and inhibitory (red). The inhibitory network controls the activity of the model. The input arrives into a particular subpopulation of all neurons. The fraction of neurons that receive input is denoted by 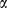 and the intensity of the external stimulation is denoted by 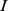. The main network parameters are the probability of connections between the neurons, 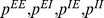 and their strength 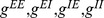. Our theoretical results indicate that the connections from the inhibitory population to the excitatory population are most important for gain control purposes.

We are investigating these conditions in the framework of a model network of excitatory and inhibitory neurons inspired by the structure of the insect AL (note similar studies in the olfactory bulb [2], [42]). Since this work is of broad relevance to brain microcircuits we will refer to the PNs as the excitatory population and the inhibitory LNs as the inhibitory population in the remainder of the paper. Excitatory LNs are included only indirectly as lateral excitatory connections between neurons of the excitatory population. Excitation from ORNs will be referred to as “sensory input”. The network architecture is illustrated in Fig. 1. As we will show below, one can use a mean field approximation to derive general gain control conditions on the connectivity of the network. We then demonstrate the validity of the mean field solution in simulations of an appropriate firing rate model and a more realistic Hodgkin-Huxley type conductance based network model.

We consider two main cases to understand gain control in the theoretical mean field model. First, we consider the situation where increasing the external stimulus increases the level of depolarization of the neurons in the network but otherwise keeps the input, in particular the number of neurons which receive input, unchanged. In the second case we consider a scenario where an increasing number of neurons is recruited (analogous to the recruitment of more glomeruli) by the stimulus when the odor concentration increases.

## Results

### Mean field description

Departing from the firing rate models explained in the model section using equations (29,30) the first step to find global conditions for robust gain control is to use mean field equations which are exact in the limit of large 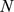. These are built by defining new macroscopic variables representing the groups of neurons depicted in Fig. 1 as
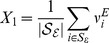
(1)

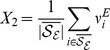
(2)

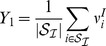
(3)

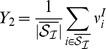
(4)The quantities 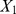 and 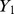 respectively represent the averaged firing rates of the excitatory and inhibitory populations, which receive sensory input for a given stimulus. We do not consider excitatory LNs explicitly but allow for excitatory connections between the neurons of the excitatory population to emulate their effects on the activity of the network. The sets 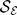 and 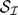 denote the indices of the excitatory and inhibitory populations.

The size of these sets can be expressed in terms of the ‘sparseness parameter’ 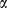 as 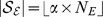 and 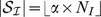, where 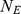 and 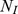 are the total number of excitatory and inhibitory neurons, respectively.

For the sake of simplicity we will assume that the external input is not fluctuating and is identical for all neurons in these sets. 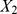 and 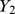 are the average activities of the excitatory and inhibitory neurons that do not receive direct input from the receptors but only laterally from the network (see Fig. 1). Their indices are denoted by 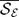 and 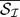.

The lowest-order mean field approximation is based on the following assumption
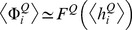
(5)where 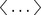 denotes a population average and the contribution of the higher order moments of the excitatory and the inhibitory synaptic currents have been considered in the vector field functions 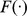. These functions are smoother than the gain functions 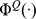 and are derived from averaging over fluctuations in the synaptic input current 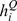 [43], see model section. Under the assumption of statistical independence between the connectivity and the activity of the network (33) and by virtue of the mean field approximation (5), we can reduce the initial microscopic field equations (29,30) to a set of four ordinary differential equations representing the average firing rate time evolution of the excitatory and inhibitory populations, separately for those that receive external input from the receptors and those that only receive input from the network.
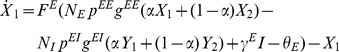


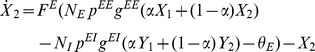


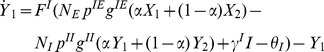


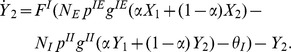
where the dot denotes the time derivative. Let us define the variables 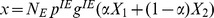 and 
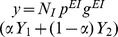
 which represent the average synaptic current from the PN and LN populations respectively. The parameter 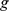 denotes the efficiency of the connection between neurons of different populations and 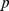 the connection probability. With these notations we can compress the four previous equations into
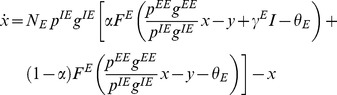
(6)

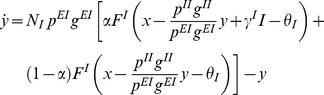
(7)Now, we first would like to prove that all the fixed points of this system of ordinary differential equations are stable and, second, to determine conditions that lead to gain control of the activity level of the excitatory population. To be more specific, we define a gain control system as a neural network with the ability to keep the averaged activity of the excitatory neurons constant over large variations in odor concentration.

#### Stability analysis

Let us first analyze the stability of the stationary state of the mean field equations. The parameter values should be set in regions where stable fixed points are feasible. Moreover, we can also identify the main source of instability which, in this case, are the excitatory connections within the excitatory population. For simplicity we define the functions
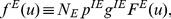
(8)

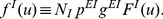
(9)The mean field equations (6, 7) are then replaced by
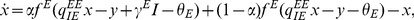
(10)

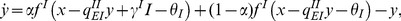
(11)where the ratio 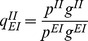 is a measure of the effective synaptic inhibition in the network, and 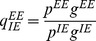 is a ratio of the effective synaptic excitation in the network. Using linear stability analysis it is easy to determine the stability conditions for the steady states of equations (10) and (11). The Jacobian of the system can be expressed as
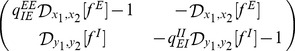
(12)where 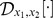 is the composite derivative operator
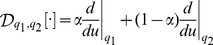
(13)and the sub-indices 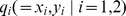 correspond to the evaluation points 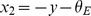, 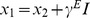, 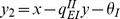, and 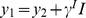. We are primarily interested in the sign of the eigenvalues of the Jacobian
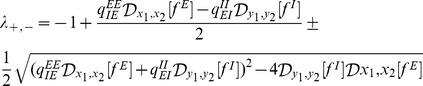
(14)In the absence of lateral excitation, 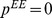 or 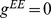, both eigenvalues are always negative because both 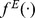 and 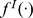 are monotonically increasing functions that implies that their derivatives 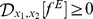 and 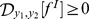. So any fixed point of the system is stable, independent of the spread of the stimulus 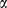. If the input is stationary, the network therefore may evolve to a solution in which the population average firing rates are constant.

However when there is some level of lateral excitation the stability conditions change for high values of the product 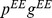. For example, for 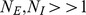 and linear gain functions with slope 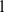, the boundary condition of stability is 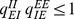, so
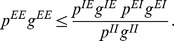
Beyond this level of lateral excitation between the neurons of the excitatory population, the dynamical system becomes unstable and non-functional for stimulus encoding purposes.

#### Gain control conditions

The first step in the analysis of equations (10) and (11) is to calculate the equilibrium firing rates of the excitatory and inhibitory populations. The equilibrium equations are found by setting 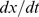 and 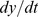 to zero, leading to
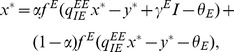
(15)

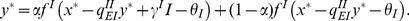
(16)
Equations (15) and (16) are nonlinear implicit equations. We need to determine conditions such that
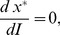
(17)that is, there are no macroscopic changes in the excitatory population activity as a function of the stimulus intensity. We are going to consider two cases. First, we consider the case where increasing intensity of the external stimulus 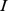 increases the level of depolarization of the neurons, while keeping the fraction 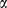 of neurons that are receiving input constant. In the second part we will consider the case where increasing odor concentration increases both the the fraction 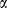 of recruited neurons and the input current 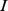 received by them [44].

#### Condition for independence on the stimulus intensity 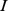


Equations (15) and (16) are implicit equations and we need to determine conditions such that 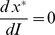. If we differentiate equations (15) and (16) we obtain
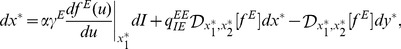
(18)

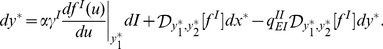
(19)where the derivative operators 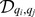 are as defined in (13) and must be evaluated at the fixed point 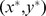. If we solve for 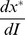 from equations (18) and (19) and then take into account the constraint (17), we obtain the gain control condition
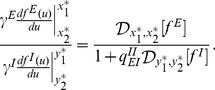
(20)This equation is one of the main results of our analysis. It describes a specific relationship between network parameters that must hold in order to maintain constant average PN activity over a large range of input intensities 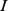. This relationship depends on the gain functions of the neurons through the slopes of the vector fields 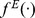 and 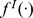 of the excitatory and inhibitory populations at the equilibrium firing rates.

#### Analysis of the gain control conditions

The general gain control condition (eq. 20) may appear complex, but it can be simplified significantly in practice. Whenever a sufficiently strong stimulus is present, and if, as we assume, lateral excitation is dominated by lateral inhibition, the group of excitatory and inhibitory neurons that do not receive direct sensory input become silent due to inhibition by the increasingly responding inhibitory neurons [32], [45]. Note, however, that this does not necessarily imply that odor responses may not broaden within the population of PNs which do receive inputs or by PNs that are additionally recruited to receive input. The gain functions of both excitatory and inhibitory neurons below threshold are constant and hence the evaluation of equation (20) at 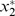 and 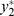 is 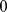 if they are silenced.

Moreover, the excitatory and inhibitory neurons that do receive sensory inputs are most of the time in the linear regime and hence the vector fields 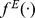 and 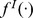 are approximately linear above their threshold [46]

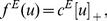
(21)

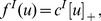
(22)where the mean input current to the excitatory population is 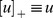 for 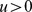 and zero otherwise, and 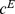 and 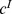 are gain parameters.

Thus, using equations (8,9,21,22), 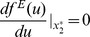, which corresponds to the group of excitatory neurons that are shut down during stimulation, 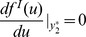, for the same reason for the LNs, and inserting them into equation (20) leads to the simple gain control expression
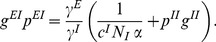
(23)Furthermore, in the simulations that follow, we use 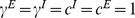, which simplifies the gain control condition to
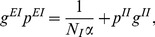
(24)indicating that the primary dependence of the gain control is on 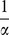. The simulation of the full set of ordinary differential equations for the firing rate and Hodgkin-Huxley models confirms this dependence as we will show in the following sections.

This expression also indicates that in order to maintain gain control, the *effective* synaptic inhibition should be scaled with the input to the network. This finding is consistent with the idea that a larger number of activated glomeruli may induce more lateral inhibition to set the appropriate sensory gain [20]. It is also remarkable that there is no explicit dependence on the connections from the excitatory to the inhibitory population, which is consistent with other findings [47]–[49] where the key plasticity is found in the connections originating from the LNs.

**Figure 2:**
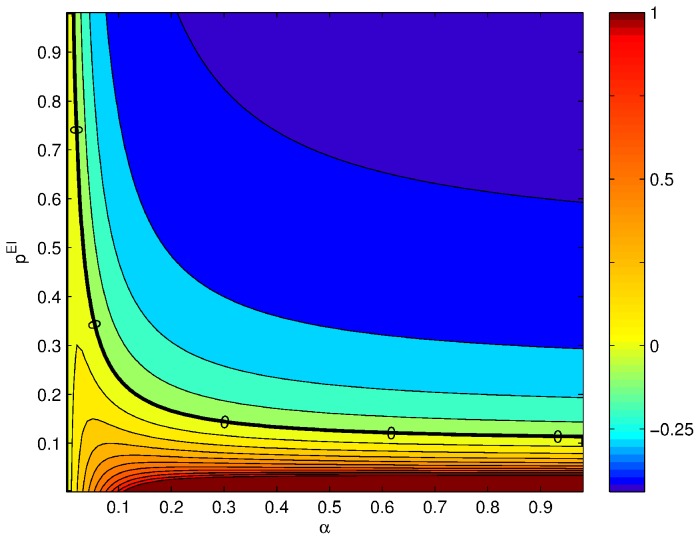
Gain control conditions obtained from the mean field approximation for 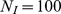, 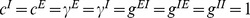, 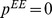, 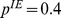 and 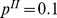. The contour plot shows the absolute value of the slope of the rate in the excitatory population as a function of the input current 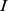 from ORNs. The axes of the plot are ranges of parameters. Strict gain control corresponds to 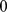 slope, eq. (23), indicated by thicker black line.

In Fig. 2 we show the derivative of the average rate of the excitatory population with respect to intensity, 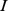, as a function of the probability of connections from the inhibitory to the excitatory population and as a function of 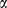. The solid black line indicates the exact gain control condition given by equation (24). This plot will be compared with the solutions obtained in the following sections using the complete firing rate and Hodgkin-Huxley network models.

Recent work in *Drosophila* [40] has demonstrated that lateral inhibition scales linearly with the total sensory input. If we calculate the fixed points of the excitatory and inhibitory populations for large 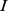 using the condition for gain control in equation (23), it can be shown that the activity of the inhibitory population scales linearly with 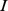 as follows
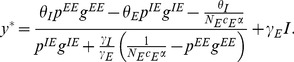
(25)Note that if the system operates in the gain control condition, the main dependence on the external input is regulated by the synaptic gain of the input to the excitatory neurons. Equation (25) implies that the activity of the inhibitory network grows linearly with the external input to compensate for the external stimulus.

#### Condition for gain control with respect to the sparseness parameter 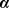


In the previous section we obtained the gain control condition for the activity of the excitatory population if the input intensity 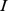 varies but the spread or sparseness parameter 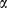 is fixed. As mentioned earlier, in general odor intensity also determines the number of recruited glomeruli and hence the equivalent of 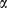 in the insect brain should depend on the stimulus as well [44]. Returning to equations (15) and (16) differentiating them with respect to 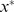, 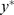, 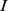 and 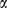 we obtain
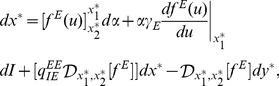
(26)

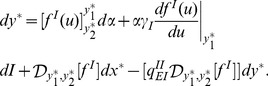
(27)When we solve these equations for 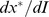 and determine the condition such that 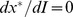, we find
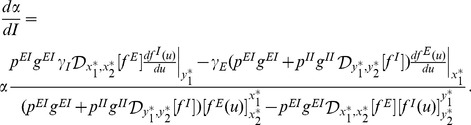
(28) This means that there is no solution to the gain control problem for arbitrary relationships between 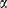 and 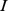. Gain control could be achieved if each change in 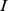 were accompanied by the correct change in 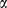 to fulfill (28). However, this would imply that this complex equation would have to be implemented in the connectivity between sensory input and the network and/or within the network or appropriate dynamic changes in the connectivity strengths 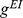 and 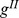 depending on 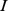, which appears unlikely.

The consequences are significant because there are no plausible gain control conditions if the stimulus is encoded with an increasing number of recruited neurons. If one looks at Fig. 2 the explicit 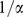 dependence shows that in order to have strict gain control conditions, the network would have to have a mechanism to modulate the probability or strength of the connections. The modulation of the inhibitory connection in real time as a function of the stimulus requires additional circuits that are not part of the mathematical description used here, although they might be possible by modifying the architecture of the network [50], [51]. If the gain control requirements can be relaxed to signify an approximately zero gain of activity with increasing input, relaxed gain control conditions can be found for a large number of inhibitory neurons and high 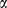. In this case the effect of changes in 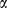 becomes negligible (see equation (24)).

### Rate model simulation

In this section we assess the validity of our approach of deriving gain control conditions from mean field approximations by numerically solving the full rate model expressed by the coupled ordinary differential equations (29) and (30) explained in the model section. The equations model the firing rate of neurons to a first approximation [52]–[55] and though they are simpler than conductance-based models, they still allow unveiling fundamental principles underlying the cooperative function of neural systems. Population rate models provide an accurate description of the network behavior when the neurons fire asynchronously [56].

**Figure 3:**
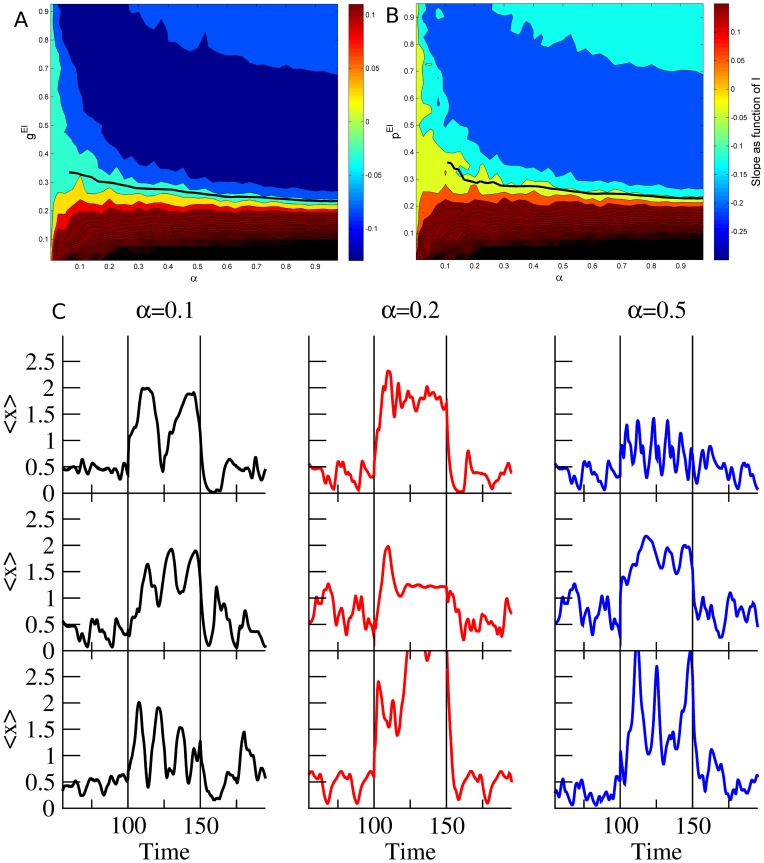
(A) Contour plot of slopes of response curves in numerical experiments using rate model neurons. We plot the derivative of change in the PN activity with respect to the external stimulus 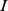 as a function of 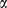, which is the fraction of neurons that receive input, and 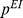, which is the probability of having a connection from an inhibitory neuron to an excitatory one. The parameter values used in this simulation of the rate model neurons were 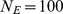, 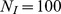, 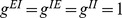, while the probability of connections are 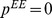, 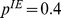 and 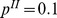. The gain constants 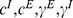 are set to 1 with thresholds 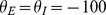. Strict gain control corresponds to 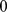 slope and is represented by the thick black solid line. The line is not complete because we do not have enough resolution to reliably track the gain control boundary. (B) Exploration of the dependence of the gain control boundaries as a function of the strength of the coupling from the excitatory to the inhibitory population. The parameters were the same as for the simulations shown in A except 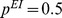. (C) Examples of the mean activity of the excitatory population for different realizations (rows) of the network using the same parameter values as (A) near the gain control conditions. Despite the restraining gain control conditions the dynamics of the rate models are capable of displaying a rich variety of dynamical behaviors. Each column represents different levels of recruitment (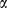) by the input.

Fig. 3 summarizes how the the steady state firing rate depends on the stimulus intensity 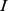. A grid of 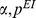 from 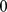 to 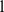 with steps of 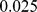 was run 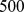 times for each range of concentrations 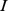. The solid thick line represents the gain control conditions, that are fairly flat for sufficiently high values of 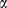 and hence match the asymptotic theoretical behavior derived in equation (24). However, for low values of 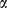, the firing rates decrease significantly as a function of 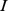 and the numerical estimation does not have sufficient precision. As we can see, there are remarkable similarities between the rate model simulation and the theoretical gain control conditions solved in equation (20) and shown in Fig. 2. In Fig. 2B we can see that the same qualitative dependence exists when the strength 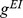 of the connections is varied rather than their probability 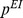. On Fig. 2C we can see a few examples of the mean activity of the excitatory population for several levels of 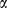 for the gain control conditions. As we can see, despite enforcing gain control the dynamics of the network retain a large repertoire of dynamical behaviors that can be used for information processing purposes.

**Figure 4:**
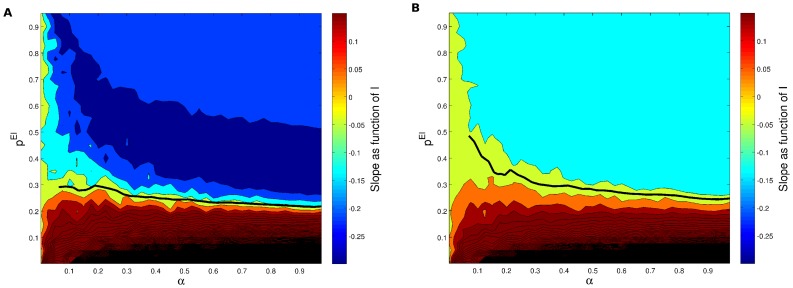
(A) Contour plot of slopes of response curves in numerical experiments using rate model neurons but here testing whether the gain control conditions are truly independent of 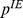 as predicted by the mean field model. We plot the derivative of change in the PN activity with respect to the external stimulus 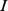 as a function of 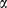, that is the fraction of neurons that receive input, and 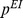, which is the probability of having a connection from an inhibitory neuron to an excitatory one. The parameter values used in this simulation of the rate model neurons were 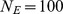, 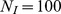, 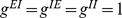, while the probability of connections are 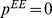, 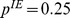 and 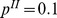. The gain constants 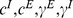 are again set to 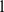 and 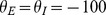. Strict gain control corresponds to 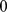 slope and is represented by the thick black solid line. The line is not complete because we do not have enough resolution to reliably track the gain control boundary. (B) The same as in the left but using 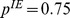 to corroborate the gain control condition (the solid line) does not depend on the connections from the excitatory population as predicted from the theory.

Overall the functional dependence of the average excitatory firing rate on the odor concentration is highly non-linear: weaker inputs from the antenna are amplified greatly, while stronger inputs are amplified less. The inhibition acts as a negative feedback loop keeping the output of the system within a given range. When we run simulations for different values of the connection probability from the excitatory to the inhibitory population, 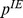, (see Fig. 4) we do not find such a high variability in the gain control conditions, which is indicated by the solid black line in Figures 4 and 3. This is again consistent with the expression (24) which lacks an explicit dependence on 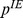 and 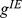.

### HH model simulation

**Figure 5:**
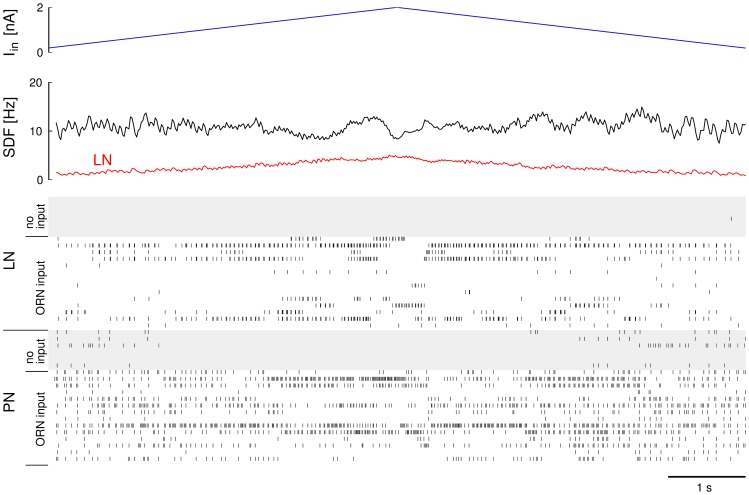
Illustration of the conductance based numerical experiment for an example of successful gain control at 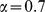 and 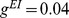 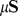. The input current to the fraction of 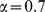 of all PNs and LNs is ramped up from 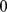 nA to 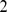 nA and back down to 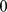 nA (top). In response to this input the firing patterns of PNs and LNs change. While the average rate of LNs increases and decreases proportional to the input (see spike density function (SDF) in the second panel), the average activity of PNs remains constant. Nevertheless, there is a clear and distinctive response to the input in form of slow patterning of the PN activity (see spike raster).

**Figure 6:**
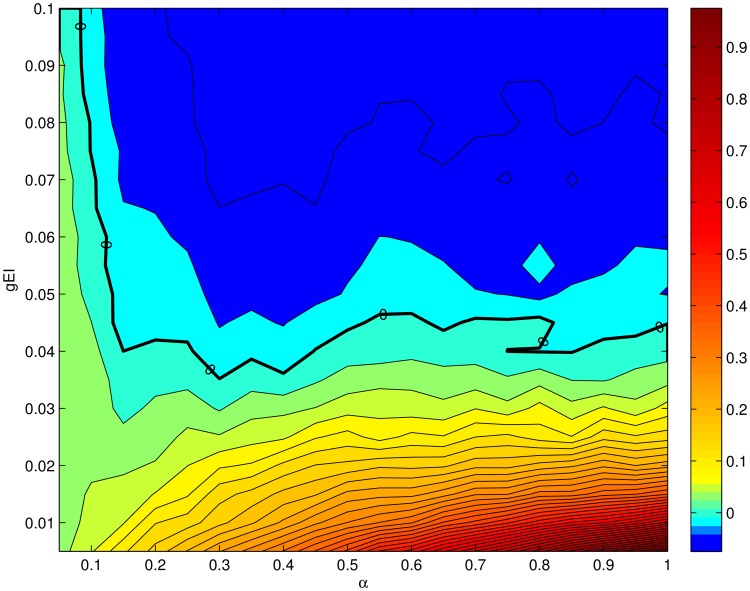
Gain with increasing 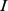 in the excitatory population as a function of 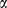 and 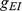 for the conductance based model. The color map shows the the slope of the spike count in the excitatory population as a function of the input current 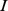 from ORNs, normalized to a maximum of 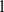. The axes of the colormap are the ranges of parameters 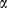 (x-axis) and 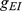 (y-axis). Strict gain control corresponds to 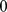 slope (green, thicker contour line).

To further test our results in an even more realistic simulation we tested the gain control condition in a network model of Hodgkin-Huxley type model neurons. We simulated a network of 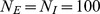 model neurons and after an initial period of 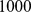 ms simulated time, we excited a fraction 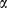 of the PN and LN populations with an input current that was ramped up linearly from 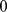 nA to 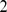 nA during 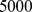 ms simulated time and then ramped down again to 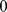 nA in another 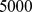 ms (Fig. 5). The input current of all excitatory and inhibitory neurons that received input was updated every integration time step. We counted the spikes in the excitatory and inhibitory population in 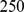 ms windows and added the numbers of the windows with corresponding input current from the up- and down-ramp. We then used Matlab to fit a linear regression to the spike count in the excitatory population as a function of the input current 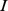. This analysis was repeated for different pairs of values for 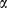 and 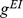. Fig. 6 summarizes the results. The slope 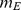 of the number of spikes 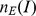 in the excitatory population in 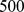 ms simulated time as a function of the input current 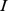 in nA (normalized to within the interval 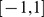) is displayed as colors. Successful gain control corresponds to 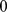 slope, or green in the plot. It is achieved on what roughly looks like a hyperbolic function 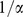 (see strong contour line). This is in good correspondence with the dependency we found in the other descriptions (see figures 2,3 and equation (23)).

Note that even though the overall activity levels are constant because of the gain control condition, Figures 3C and 5 illustrate that nevertheless the network does produce a variety of spatio-temporal responses of the excitatory population. Gain control is therefore not limiting the capacity to map external information into intrinsic neural representations. It is known that networks of excitatory-inhibitory neurons, like the one used here, can achieve a large repertoire of reproducible spatio-temporal sequences to encode information [57].

## Discussion

The function of gain control is necessary if not crucial for any system that aims to separate the quality of stimuli from their intensity. If this separation is achieved there must be a stage in the signal processing system where the response is no longer dependent on the intensity of the signal. This has been observed in biological systems and is believed to be important for the correct function of neural systems [1]. Mechanisms of gain control have been demonstrated at the level of single neurons using, e.g., synaptic adaptation [51]. It was found that adapting synapses allowed signal decoding over a wide dynamical range even though it did not induce signal invariance per se. Another commonly suggested mechanism of gain control is feedback gain control [29] which has been found to effectively stabilize general activity levels independent of stimulus intensity and so support efficient coding of odor identity independent of concentration [41].

Besides being important for separating intensity and identity information, models of the insect brain have demonstrated that gain control is also an important constraint for improving recognition performance [21], [58], [59]. For example in the insect olfactory system, the excitatory neurons of the AL project into a large screen of mushroom body neurons where there is a large variability of activity as a function of small perturbations in the AL [28], [30], [58]. It is therefore imperative to closely control activity levels in the AL in order to have usable sparse coding in the mushroom bodies.

Here, we demonstrated gain control at the level of a subnetwork. Gain control is achieved through a balance between excitation and inhibition, while both excitatory and inhibitory neurons in the system receive excitatory input from primary sensory neurons. Although the neurons in the network are using constant synaptic connections, i.e., are lacking synaptic adaptation, we were able to identify successful gain control conditions. The mechanism that underlies these conditions emerges from the dynamic balance of inhibition and excitation.

Our main finding, obtained by mean field analysis and confirmed by more detailed simulations, is that gain control conditions exist over a defined range of connectivity strengths from excitatory to inhibitory neurons if stimuli of different intensity affect the intensity of stimulation and not the number of neurons that are activated.

The success of gain control is largely determined by the probability and strength of inhibitory (LN) to excitatory (PN) connections. The strength of the connections from excitatory to inhibitory neurons, which is important for odor coding in insects [13], [60], does not play a role in the proper function of this gain control mechanism. We have also investigated the role of lateral excitation that has been found in *Drosophila* in the form of excitatory LNs [10], [61] and found that it also did not play a role in the effectiveness of gain control. These results are consistent with previous work that explored unsupervised learning in the AL network and found that LN to PN plasticity is most effective in generating olfactory habituation in the fruit fly [48], [49] and honeybee [47]. Moreover, these ideas seem to resonate with the observation that lateral inhibition on PNs narrows the glomerular response profile [32], [45], similar to the ideas proposed in the olfactory bulb of mammals [42].

In the insect olfactory system, when an odorant stimulus increases in strength, both the intensity of the ORN response and the number of different types of ORNs that respond increase [33]. In our model a change of the intensity of the ORN responses would be equivalent to an increase in 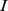. We have demonstrated that we can derive a general gain control condition for changes in 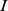 that depends only on the connections from the inhibitory population to the rest of the network regardless of the number of neurons. This gain control condition derived from the mean field approximation is valid for random networks of excitatory and inhibitory neurons using rate models and realistic conductance based models, demonstrating the generality of the result. Furthermore, in agreement with experimental evidence [40], we found that in order to achieve gain control the activity of the inhibitory population and hence the strength of lateral inhibition needs to scale linearly with the intensity of the input.

However, if 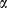, the fraction of activated glomeruli, is the main variable that encodes stimulus intensity, we could not identify consistent or stationary gain control conditions, in particular for low values of 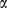. If increasing stimuli recruit a larger number of glomeruli and hence neurons, the network parameters (the probability and strength of the connections) have to change dynamically in order to regulate the activity levels of the excitatory population. Short term depression of synapses and spike rate adaptation in neurons could be invoked as possible mechanisms [50]. It is in particular unclear whether such mechanisms would be fast enough for efficient gain control and whether they would compromise the sensitivity of the network to subsequent low-intensity inputs.

A different solution to the problem of input dependent 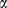 would be the relaxation of the gain control condition. We have in this paper concentrated on strict gain control by postulating 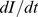 to be exactly 0 leading to exact conditions on connection probabilities and connection strengths. This is unlikely to be precisely realized in biological networks. The most plausible scenario is that the neural networks in the brain have large parameter spaces in which information processing is not impaired. Within this scenario, one would expect gain control to be approximate rather than strict. It remains to be seen how much our gain control conditions could be relaxed. Perhaps, a reasonable approach to this question may consist of determining a lower and upper bound of 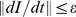 instead of equality to 0. In this case a large number of inhibitory neurons, for example, could shift the gain control conditions more aggressively to the left in Figures 2 and 3, effectively achieving very good regulation of excitatory activation for many values of 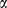. Furthermore, as the gain control curves are approximately horizontal for 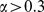, constant values of the connectivity probabilities and strengths could lead to approximate gain control in this regime even if 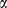 is input dependent.

The fact that the gain control conditions derived from the mean field approximation were verified by simulations of a rate model [56] and a more realistic Hodgkin-Huxley conductance based model is an important confirmation that using mean field approximations to understand the structural organization of brain centers is useful. Our formulation of the mean field theory has been proven to be general enough to capture the main function of the system. We would like to interpret this finding to indicate that the main properties of the system we have described do not critically depend on the details of its construction. In this sense, there is a large space of neural circuits with properties similar to the ones observed here.

The confirmation of the gain control conditions in a firing rate model is also important in the context of presynaptic inhibition which has been identified in several forms in the AL[11]. Firing rate models accommodate presynatic inhibition alongside postsynaptic forms of inhibition because all synaptic inputs are integrated in a passive manner. Being confirmed in a firing rate model, our gain control conditions should be valid for any form of synaptic inhibition in any combination.

In conclusion, we used analysis and simulations of a network of excitatory and inhibitory neurons inspired by the AL network to identify a relationship between network parameters that allows strict gain control. The more general question is how such a relationship can be induced and maintained in a biological system. Certainly, strict gain control would necessitate the probability of connections between the population of neurons and the strength of these connections to have a very precise value. This is, as we have already alluded to above, impractical in real world conditions. However, our simulations suggest that there is a range of values around the strict gain control condition line where approximate gain control is achieved. Therefore, a biological mechanism that would control the probability and strength of excitatory to inhibitory connections [62], [63] within a certain range would suffice to achieve the desired approximate intensity invariance.

## Models

### The firing rate network model

To analytically address the issue of *gain control* in a random excitatory-inhibitory network, we simulate the network by firing rate models [64]. Rate models [54], [55] are simpler than conductance-based models, but they reveal some of the fundamental principles that underlie the cooperative function of neural systems by providing an accurate description of the network behavior when the neurons fire asynchronously [56].

The network mode l considered here consists of an excitatory PN population, and an inhibitory LN population with 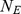 and 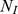 neurons respectively. We use the sub- or superscript 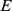 for variables referring to the excitatory population (PNs) and 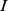 for those referring to the inhibitory population (LNs). The firing rates of all neurons evolve in time according to the following set of ordinary differential equations:
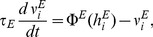
(29)

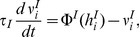
(30)where 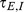 are the time constants, 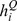 denotes the total afferent current into the 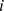
th neuron in pool 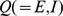 and 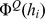 is the corresponding gain function. The individual gain functions represent the steady state firing rates of the neurons as a function of their total input. Note that we do not consider excitatory LNs at this level of description.

To be as general as possible we only make two assumptions about the model neurons. First, that they have a threshold, i.e.,
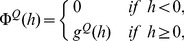
(31)and second, that their gain functions are positive, continuous and monotonically increasing functions (i.e., 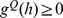 and 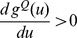). These conditions are quite general and represent fairly well the firing response of neurons [65]. Furthermore, specific gain functions can be determined semi-analytically for specific noise models or numerically for more realistic models [56], [66], [67].

The neurons are connected through a network of synaptic connections 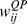. The contributions of all synapses are assumed to be added linearly in the main compartment of the neuron via
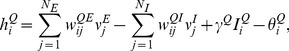
(32)where 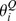 is the threshold of the 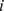
th neuron in pool 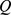 and the term 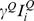 represents its external input from the presynaptic ORNs. Both, PNs and LNs, receive afferent input directly from the ORNs.

In our model we do not assume any anatomically or functionally structured connectivity between the glomeruli, i.e., the connectivity matrices 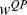 are random matrices with entries drawn from the following Bernoulli process
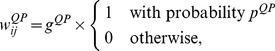
(33)where 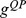 represents the synaptic conductance or efficiency of the connection from a neuron in the pool 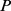 to a neuron in the pool 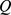. Thus, on average, a given neuron receives 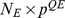 synapses of strength 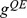 from the excitatory population and and 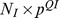 connections of strength 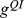 from the inhibitory population.

### The Hodgkin-Huxley network model

The simulated network of conductance based Hodgkin-Huxley neurons consists of 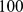 PNs and 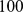 LNs which were randomly connected as described for the rate model above. The probabilities for connections were 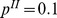 following observations in the honeybee [45], 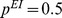 and 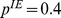, and 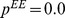.

### HH neuron model

The neurons in the simulated network model were described by a Hodgkin-Huxley type model based on the model of Traub and Miles [68]. Additionally, a spike rate adaptation (M-type) current was added leading to the following set of equations:
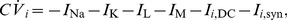
(34)where 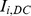 is a constant bias current regulating the intrinsic excitability of neurons. The leak current is 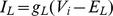 and the ionic currents 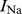, 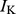, and 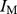 are described by
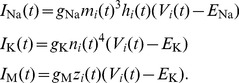
(35)The synaptic current 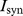 to each neuron is the linear sum of all synapses onto the neuron, each synaptic current given by (38). Each activation and inactivation variable 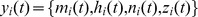 satisfied first-order kinetics
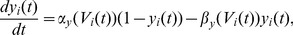
(36)with non-linear functions 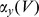 and 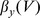 given by
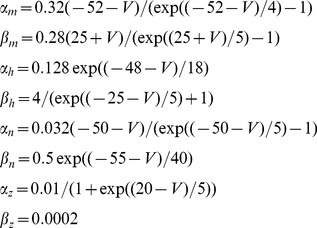
(37)The remaining parameter values were 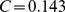 nF, 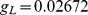 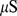, 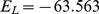 mV, 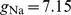 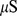, 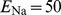 mV, 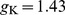 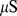, 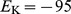 mV, 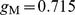 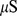. 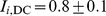 nA for PNs and 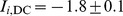 nA for LNs, where 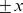 denotes the addition of a random individual bias sampled from a uniformly distributed random variable in 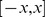 for each neuron.

### Synapse model

Synapses were described by a first order kinetic model [69]. In brief, the synaptic current is given by
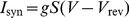
(38)where 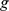 is the synaptic conductance and 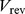 the reversal potential. Here, 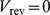 mV for excitatory synapses and 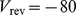 mV for inhibitory synapses. 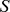 describes the activation of the synapse and is governed by
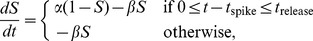
(39)where 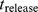 is the duration of synaptic release after a pre-synaptic spike, 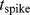 is the time of the last pre-synaptic spike, detected as a crossing from below of 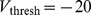 mV, and 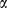 and 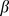 are rates of synaptic release and decay (re-uptake). Here, we used 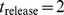 ms for excitatory synapses and 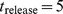 ms for inhibitory synapses. The activation and inactivation rates were given by 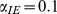 kHz, 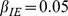 kHz, 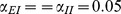 kHz and 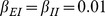 kHz. The maximal synaptic conductances were chosen as 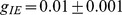 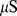, 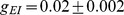 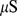, and 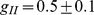 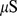, where 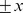 denotes a random variation by a Gaussian random variable of mean 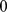 and standard deviation 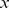. The model was integrated with a 6/5 order variable time step Runge-Kutta algorithm with maximal time step of 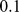 ms, using custom-made C++ code.
